# Near-Roadway Air Pollution and Coronary Heart Disease: Burden of Disease and Potential Impact of a Greenhouse Gas Reduction Strategy in Southern California

**DOI:** 10.1289/ehp.1408865

**Published:** 2015-07-07

**Authors:** Rakesh Ghosh, Frederick Lurmann, Laura Perez, Bryan Penfold, Sylvia Brandt, John Wilson, Meredith Milet, Nino Künzli, Rob McConnell

**Affiliations:** 1 Department of Preventive Medicine, Keck School of Medicine, University of Southern California, Los Angeles, California, USA; 2 Sonoma Technology Inc., Petaluma, California, USA; 3 Swiss Tropical and Public Health Institute, Basel, Switzerland; 4 University of Basel, Basel, Switzerland; 5 University of Massachusetts Amherst, Amherst, Massachusetts; 6 Spatial Sciences Institute, Dana and David Dornsife College of Letters, Arts, and Sciences, University of Southern California, Los Angeles, California, USA; 7 California Department of Public Health, Richmond, California, USA

## Abstract

**Background:**

Several studies have estimated the burden of coronary heart disease (CHD) mortality from ambient regional particulate matter ≤ 2.5 μm (PM_2.5_). The burden of near-roadway air pollution (NRAP) generally has not been examined, despite evidence of a causal link with CHD.

**Objective:**

We investigated the CHD burden from NRAP and compared it with the PM_2.5_ burden in the California South Coast Air Basin for 2008 and under a compact urban growth greenhouse gas reduction scenario for 2035.

**Methods:**

We estimated the population attributable fraction and number of CHD events attributable to residential traffic density, proximity to a major road, elemental carbon (EC), and PM_2.5_ compared with the expected disease burden if the population were exposed to background levels of air pollution.

**Results:**

In 2008, an estimated 1,300 CHD deaths (6.8% of the total) were attributable to traffic density, 430 deaths (2.4%) to residential proximity to a major road, and 690 (3.7%) to EC. There were 1,900 deaths (10.4%) attributable to PM_2.5_. Although reduced exposures in 2035 should result in smaller fractions of CHD attributable to traffic density, EC, and PM_2.5_, the numbers of estimated deaths attributable to each of these exposures are anticipated to increase to 2,500, 900, and 2,900, respectively, due to population aging. A similar pattern of increasing NRAP-attributable CHD hospitalizations was estimated to occur between 2008 and 2035.

**Conclusion:**

These results suggest that a large burden of preventable CHD mortality is attributable to NRAP and is likely to increase even with decreasing exposure by 2035 due to vulnerability of an aging population. Greenhouse gas reduction strategies developed to mitigate climate change offer unexploited opportunities for air pollution health co-benefits.

**Citation:**

Ghosh R, Lurmann F, Perez L, Penfold B, Brandt S, Wilson J, Milet M, Künzli N, McConnell R. 2016. Near-roadway air pollution and coronary heart disease: burden of disease and potential impact of a greenhouse gas reduction strategy in Southern California. Environ Health Perspect 124:193–200; http://dx.doi.org/10.1289/ehp.1408865

## Introduction

Emerging evidence suggests a causal link between near-roadway air pollution (NRAP) and coronary heart disease (CHD) mortality and morbidity (Gan et al. 2010, 2011; Hoffmann et al. 2006; Kan et al. 2008). The 2010 American Heart Association scientific statement on ambient particles noted that NRAP “as a whole appears to be a specific source associated with cardiovascular risk” (Brook et al. 2010). Since then, additional longitudinal studies have demonstrated consistent associations between NRAP and CHD, using traffic density, proximity to roadways, and a near-roadway pollutant surrogate, elemental carbon (Gan et al. 2010, 2011; Kan et al. 2008). Although the specific pollutants in NRAP responsible for health effects are not entirely clear, evidence suggests that NRAP effects are independent of those of particulate matter ≤ 2.5 μm (PM_2.5_) (Hoffmann et al. 2006). However, in contrast to PM_2.5_, there has been little examination of the NRAP-attributable disease burden. Furthermore, although regional PM levels have been declining in most of the United States over several decades (Motallebi et al. 2003) due to effective regulatory policy, some indicators of NRAP exposure such as vehicle miles traveled have increased markedly over the same period (U.S. Department of Transportation 2013). There is a need to assess the NRAP-attributable burden of disease.

We assessed the burden of CHD attributable to NRAP relative to PM_2.5_ in Southern California, which has high regional PM_2.5_ levels and a dense network of high-volume traffic corridors in close proximity to residences. We also estimated the CHD health co-benefits of California’s landmark legislation (SB 375) to reduce greenhouse gas emissions (more than one-third of which come from cars and trucks) by 16% in 2035. The Southern California Association of Governments (SCAG) has developed a regional plan that aims to reduce per capita vehicle miles traveled, because this has substantial impact on greenhouse gas emissions (SCAG 2012a). This is to be accomplished with a land use development strategy designed to reduce the need for automobile travel by encouraging denser residential development in already developed urban areas that are served by public transport and by discouraging new development in currently undeveloped areas (SCAG 2012a). To support compact urban development conducive to walking and use of public transportation, transportation investment will focus on improving public transport by increasing service frequency and transit connections, and creating bicycle and pedestrian infrastructure. The California Air Resource Board’s and the U.S. Environmental Protection Agency’s (EPA) stricter vehicle exhaust emission standards, requirements for increased proportions of zero emission vehicles, and higher fuel economy standards are expected to substantially reduce future conventional and greenhouse gas emissions per mile of vehicle travel. We estimated the population exposure to NRAP and PM_2.5_, which will be associated with implementation of these changes, and the corresponding pollution-attributable CHD.

## Methods

*Concentration–response functions*. There are only a few studies of associations of CHD mortality and hospitalization with NRAP conducted in North America and therefore more likely to be relevant to Southern California than studies from other parts of the world. We used concentration–response functions (CRF) from studies of two surrogates of NRAP exposure: traffic density and residential proximity to a major road (Table 1). The traffic density CRF was based on a four-communities study in the Midwestern and Eastern United States (Kan et al. 2008). We used a CRF for residential elemental carbon (EC), based on black carbon, derived from an administrative data set covering the entire Vancouver, Canada, population (Gan et al. 2011). (For estimating EC-attributable burden of disease, black carbon was converted to EC, as described in the Supplemental Material, “Methods.”) EC is an indicator of diesel exhaust exposure in Southern California (Geller et al. 2005) and is commonly considered a near-roadway pollutant (Wu et al. 2009). EC may provide a lattice for toxicologically relevant metals and adsorbed organics that are inhaled deep into the lung (Bell et al. 2009; Janssen et al. 2012). We selected the CRF from the Vancouver study, because it was estimated from a network of measurements reflective of fine-scale spatial variation heavily influenced by roadway sources, and was derived for a similar age distribution and for CHD outcomes comparable to the CRFs for other NRAP indicators used in this analysis. For consistency, we used a CRF for proximity to a major road derived from the Vancouver study (Gan et al. 2010). For comparison with the NRAP effects, we also estimated the burden of regional PM_2.5_ exposure, based on a CRF that is used in mortality risk assessment for regulatory purposes by the U.S. EPA (Krewski et al. 2009; U.S. EPA 2009).

**Table 1:** Study characteristics and the concentration–response functions (CRF) used in the attributable fraction estimation.

Study characteristics	Kan et al. 2008	Gan et al. 2010	Gan et al. 2011		Krewski et al. 2009
			Hospitalizations	Mortality	
Geographic area	Forsyth, NC; Jackson, MS; Minneapolis, MN; Washington, MD; USA	Vancouver, Canada	Vancouver, Canada	Vancouver, Canada	USA (nationwide)
Study year	Recruitment 1987–1989, Follow-up through 2002	5-year exposure (1994–1998), 4-year follow-up (1999–2002)	5-year exposure (1994–1998), 4-year follow-up (1999–2002)	5-year exposure (1994–1998), 4-year follow-up (1999–2002)	Exposure 1999–2000, follow-up 1982–2000
Mean age (± SD), range (years)	55.8 ± 5.6 45–64	58.7 ± 10.4 45–83	58.7 ± 10.4 45–83	58.7 ± 10.4 45–83	56.6 ± 10.5
Exposure	Traffic density count per day^*a*^ (per 1 log unit)	Residence ≤ 150 m from a highway or ≤ 50 m from a major road compared with all others	Black carbon^*a*^ (per 0.94 × 10^–5^/m)	Black carbon^*a*^ (per 0.94 × 10^–5^/m)	PM_2.5_ (per 10 μg/m^3^)
*n* (cases)	13,309 (976 deaths)	414,793 (3,133 deaths)	452,735 (10,312 hospitalizations)	452,735 (3,104 deaths)	488,370 (29,989 deaths)
Outcome	Myocardial infarction/coronary revascularization/CHD death^*b*^	CHD mortality^*c*^	CHD hospitalizations^*c*^	CHD mortality^*c*^	CHD mortality^*d*^
CRF^*e*^ (95% CI)	1.03 (mortality) (1.01, 1.05)	1.29 (mortality) (1.18, 1.41)	1.03 (hospitalization) (1.01, 1.05)	1.06 (mortality) (1.03, 1.09)	1.15 (mortality) (1.13, 1.20)
^***a***^Traffic density values were proportional to proximity-weighted vehicles per day where one density unit corresponded to 295 vehicles per day at 10 m from the roadway. It declines linearly with distance to zero vehicles per day at 300 m from the roadway. Black carbon scaled to interquartile-range increase in absorbance. ^***b***^ICD-9 codes 402, 410–414, 427, 428, 518.4; ICD-10 codes E10–14, I10–11, I21–25, I46–51, I70, I97, J81, J96, R96, R98–99. ^***c***^ICD-9 codes 410–414, 429.2; ICD-10 codes I20–I25. ^***d***^ICD-9 codes 410–414. ^***e***^Estimates are hazard ratios (95% CIs), which were scaled to the population-weighted mean exposures for 2008 and 2035 and used in the attributable fraction calculation.					

**Figure 1:**
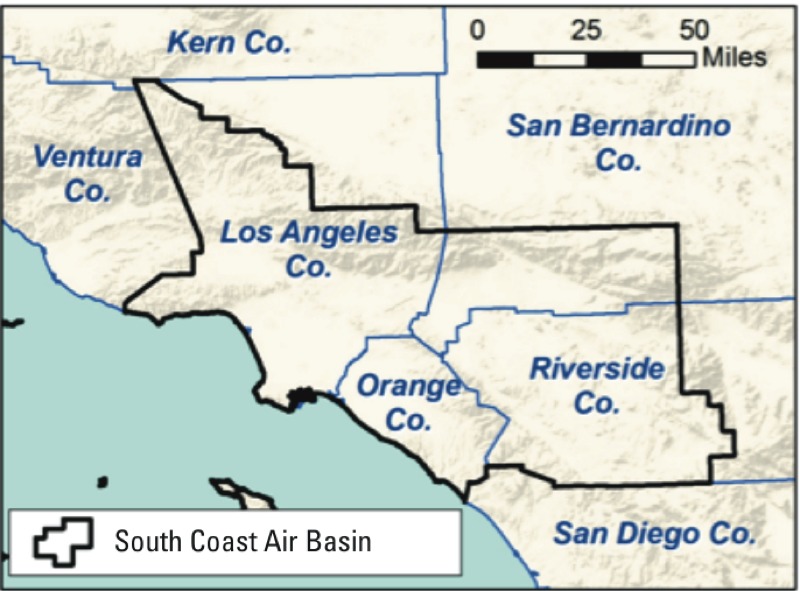
Geographical coverage of the study area is shown by the thick black border. Thin blue lines show the county boundaries and the coastline.

*Population data spatial allocation*. The geographic domain for our study was California’s South Coast Air Basin (SoCAB), comprising the southern part of Los Angeles County, western portions of Riverside and San Bernardino counties, and all of Orange County (Figure 1), a region with historically high air pollution levels. Data for the total population, households, land use, and boundary polygons of the legally defined real estate parcels were acquired from the regional planning agency, SCAG, for 2008. The population and household data were spatially resolved in approximately 11,000 travel activity zones (TAZs) that are used in the agency’s travel demand models (SCAG 2012b). The TAZ populations were assigned to residential-zoned parcels within each TAZ. If all parcels within a TAZ were single-family residences, the population per household was assigned uniformly. If all parcels within a TAZ were multi-family residences, the parcel populations were apportioned based on parcel areas. If both existed, the single-family residence parcels were assigned the county-average number of persons per household, and the remainder of the TAZ population was assigned based on the areas of the multi-family parcels. The population was assumed to reside at the centroid of the land parcel, which is more accurate than traditional methods of locating population at census-block centroids or block-group centroids.

We estimated the 2035 population distribution based on the 2035 scenario of the Sustainable Communities Strategy of the regional transportation plan that was designed to maximally reduce greenhouse gas emissions in Southern California (SCAG 2012a). The population and number of households by TAZ were acquired from SCAG along with the General Plan land use for future development areas (SCAG 2012a). The population assignment method for existing parcels was the same for 2035 as 2008. To avoid assigning large populations to the center of large areas designated for future residential growth in the General Plan, we used a grid-like approach to define potential new parcels near existing and future roadways, and to apportion the future population to these parcels. The result of this procedure was total population estimates for about 4 million existing and potential new parcels in the SoCAB in 2008 and 2035.

Because the epidemiological studies of effects of air pollution on CHD were consistently conducted on the population ≥ 45 years of age, we estimated the 2008 and 2035 parcel populations in this age group using the relative age distributions from the 2010 Census tract data and 2035 county-level projection, respectively, obtained from the California Department of Finance (2013).

*CHD mortality and hospitalization*. Cause-specific mortality and hospitalization for 2008 were available by ZIP code from the California Department of Public Health by age group (45–54, 55–64, 65–74, 75–84, and ≥ 85 years). Deaths in *International Classification of Diseases, 10th Revision* (ICD-10) codes I20–I25, based on those used in the studies from which the EC and proximity to a major road CRFs were derived (Gan et al. 2010, 2011), were used to estimate CHD mortality rates for the population aggregated to the ZIP code level. We used these same ICD outcomes and rates in estimating the traffic density–attributable deaths, even though the CRF for traffic density was obtained from a study that included additional ICD codes (E10–14, I10–11, I46–51, I70, I97, J81, J96, R96, R98–99) (Kan et al. 2008). Although using the reduced number of ICD codes likely resulted in underestimated traffic density–attributable CHD deaths, it made it possible to compare the traffic density estimates with those for EC and proximity to major road. Hospitalizations for ICD-9 (*9th Revision*) codes 410–414 and 429.2 were used to calculate CHD hospitalization rates (Gan et al. 2011).

Because the projected 2035 age distribution was available only at the county level, the 2008 age-specific mortality and hospitalization rates were aggregated to the county level and applied to the projected 2035 age-specific population in each SoCAB county to estimate the corresponding death and hospitalization counts (and rates in the ≥ 45-year age group) in 2035. Because SoCAB comprises only a portion of some counties, this calculation assumed that the projected 2035 population age distribution for the geographic portion of each county in the SoCAB will be the same as that of the entire county. The estimates of mortality and hospitalization also assumed that the age-specific rates in 2008 will be the same in 2035.

*Exposure assessment*. The approach for exposure assessment involved characterization of near-road exposures using traffic density and traffic proximity markers and applications of regional- and local-scale air quality dispersion models to estimate parcel level annual average EC and PM_2.5_ mass concentrations. Regional exposure across Southern California was estimated using the Community Multiscale Air Quality model, version 4.7.1 (http://www.epa.gov/scram001/) (Carter 2000), and the Weather Research and Forecasting model version 3.3 meteorological fields (http://www.wrf-model.org/). The model analyses were conducted for a large Southern California domain extending from 160 km west of the port of Los Angeles to the Colorado River in the east, and from Bakersfield in the north to 100 km south of San Diego in the south. Model simulations were run by the South Coast Air Quality Monitoring District as part of the Air Quality Management Plan (SCAQMD 2013). The domain was spatially resolved using 4 km × 4 km horizontal grids and 18 vertical layers. Model simulations were run by the South Coast Air Quality Monitoring District as part of the Air Quality Management Plan (SCAQMD 2013). Annual conditions were simulated for a 2008 baseline and for 2035 with the regional transportation plan elements (SCAG 2012a). The emissions and meteorological inputs, modeling procedures, outputs, and model performance are described elsewhere [SCAQMD (2013), Appendices V and VI]. The regional model’s gridded estimates for annual average EC and PM_2.5_ mass concentrations were assigned to all parcels with centroids within each 4 × 4 km grid.

Because regional models cannot resolve local pollutant gradients near roadways, a line source dispersion model, Caline4 (Benson 1992), was applied to characterize the local-scale impacts of on-road mobile source EC emissions from roads within 2 km of each parcel. The Caline4 model’s estimates of annual average EC incremental concentrations from local roadway sources were superimposed on the regional model estimates for each parcel. The Caline4 model was applied using local surface wind data from the nearest monitoring station, light-duty and heavy-duty vehicle emission factors from the EMFAC2011 model (CARB 2011, 2013b), and roadway geometry and annual average traffic volumes from the SCAG travel-demand model.

The SCAG travel-demand model for roadways was used to simulate traffic for the 2008 baseline and 2035 future scenario with the regional transportation plan control measures (SCAG 2012b). The model uses geographically accurate roadway locations for freeways and expressways (group 1), major arterials (group 2), and minor arterials and major collectors (group 3). Each travel direction was represented separately for large roads, and the smaller roads were bidirectional. SCAG developed separate traffic demand models and traffic volumes for light-duty and heavy-duty vehicles on all roadway links. Average daily traffic volumes were determined by aggregating the simulated traffic volumes for morning, midday, afternoon, evening, and nighttime traffic. SCAG applied the models to simulate traffic for the 2008 baseline and 2035 future year with the regional transportation plan control measures. The estimated future emission inventory included growth and emission controls based on the South Coast Air Quality Monitoring District’s Air Quality Management Plan (SCAQMD 2013) and SCAG’s regional transportation plan (SCAG 2012a).

Other exposure markers were the distance to nearest roads and traffic density. The distances from the center of each residential parcel to the nearest road in groups 1–2 (freeway or major arterial) were computed using ESRI’s ArcGIS tools. This is consistent with the CRF corresponding to the distance to freeways or major roads marker (150 m from the closest freeway or 50 m from the closest major road) (Gan et al. 2010).

The traffic density marker represents distance-decayed annual average daily traffic volume surrounding each residential parcel location. The SCAG roadway geometry and link-based traffic volumes were used with a ArcGIS density function that linearly decayed traffic volumes from 100% at the roadway centerline to 10% at 300 m perpendicular to the roadway. This decay rate is consistent with the observed primary pollutant concentration gradients near roadways (Karner et al. 2010; Zhu et al. 2002, 2006). The traffic density beyond the 300-m radius buffer was assigned a value of zero. Because the marker was initially developed for CHD and traffic density CRF in 1987–1989 (Kan et al. 2008), and vehicle emission rates per kilometer of travel have declined substantially since this time period, the traffic density marker was adjusted based on the EMFAC2011 model (CARB 2013a) estimates of the changes in fleet average PM_2.5_ emission rates between 1989 and 2008 (–62.1%) and projected for 2035 (–76.4%).

Using the modeled exposures for each of the three continuous exposures (traffic density, EC, and PM_2.5_), the population-weighted mean exposure was calculated by multiplying the population ≥ 45 years of age in each parcel with the exposure assigned to that parcel (p_i_). The summation of this product over all parcels was divided by the total population, as shown in Equation 1 (by county and for the entire SoCAB).


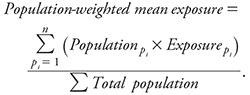
[1]

*Attributable burden estimation*. For the population ≥ 45 years, we estimated the CHD population-attributable fraction (PAF) due to residential proximity to major roadways in 2008 and 2035 based on the proportion exposed (p_exp_) and the corresponding CRF from the original study, in the standard PAF formula (Equation 2).

PAF = **p_exp_** (CRF – 1)/ [**p_exp_** (CRF – 1) + 1]. [2]

Traffic density, EC, and PM_2.5_ CRFs (Table 1) were originally reported per 1 log unit (proximity-weighted vehicles per day), per 1 interquartile range (IQR = 0.94 × 10^–5^/m of black carbon reflectance), or per 10 μg/m^3^, respectively. The population-weighted mean exposure estimated using Equation 1 was divided by the respective IQR (EC) or 10 μg/m^3^ (PM_2.5_) and this value was used to rescale the CRF to the population-weighted mean value by exponentiation (Equation 3). EC (micrograms per cubic meter) was converted to black carbon (10^–5^/m) to match with the original CRF. (See Supplemental Material, “Methods.”)

CRF_population-weighted mean exposure_ = (CRF_per unit exposure_)^population-weighted mean exposure^. [3]

Because the PAFs for traffic density, EC, and PM_2.5_ were calculated for a population-weighted mean exposure for the entire population, the proportion exposed (p_exp_) in Equation 2 becomes unity and Equation 2 reduces to Equation 4:

PAF = (CRF – 1)/CRF. [4]

We selected a background level above which the impact was quantified. For EC and PM_2.5_, PAFs were estimated for the reduction of the population-weighted mean levels to background levels of 0.12 and 5.6 μg/m^3^, respectively, based on measurements in a clean Central California coastal community (Lompoc) for the period 1994–2001 (Peters et al. 2004). Previous studies used similar background levels and methodology (Anenberg et al. 2010; Evans et al. 2013). Because traffic is entirely anthropogenic, the background level for traffic density was 1.0, as increased CHD risk (Table 1) was only observed at exposures > 1 (log traffic density of zero).

The 2008 and 2035 attributable numbers were estimated by multiplying the population ≥ 45 years by the CHD mortality or hospitalization rates and the PAF (Equation 5).

Population-attributable number_mortality/hospitalization_ = Population_≥ 45_ × Rate_mortality/hospitalization_ × PAF_mortality/hospitalization_. [5]

We calculated the PAF and the attributable number for the portion of each county within the SoCAB and also for the entire SoCAB region. The PAF and the attributable number for the distance to roadways marker of NRAP exposure can be interpreted as the proportion and number of deaths, respectively, that could be prevented if no one lived within 150 m from a freeway or 50 m from a major road. For EC and PM_2.5_, the PAF (or number of attributable events) can be interpreted as the proportion (number) that could be prevented if the population-weighted mean exposures were reduced to background levels.

To distinguish the impact of the projected change in exposure in 2035 from the impact of the projected change in the population age distribution in 2035, we estimated the attributable events for 2035 for a hypothetical scenario in which the 2008 age distribution were applied to the 2035 population.

*Statistical uncertainty analysis*. We constructed the 95% uncertainty interval (UI) around the point estimates accounting for the uncertainty in each of the parameters used to calculate the PAF, as suggested by Greenland (2004). The UI for the traffic density, EC, and PM_2.5_ PAF was calculated by incorporating the uncertainty of the rescaled CRF, that is, the hazard ratio exponentiated to the population-weighted mean. The UI for the proximity PAF was estimated accounting for the uncertainty in both parameters (proximity CRF and the proportion exposed).

## Results

The total SoCAB population was 15.5 million in 2008 and is projected to increase by approximately 3 million in 2035. However, the proportion ≥ 45 years at risk for CHD is expected to increase from 35% in 2008 to 43% in 2035 (Table 2). As a result, the increase in the CHD mortality rates, which reflect the change in the population age distribution, are projected to increase disproportionately with the population increase, from 3.4 to 4.9 deaths per 1,000 population. SoCAB CHD hospitalization rates are projected to increase from 8.9 per 1,000 in 2008 to 11.3 per 1,000 in 2035.

**Table 2:** Population ≥ 45 years and coronary heart disease (CHD) mortality and hospitalization rates overall for the South Coast Air Basin and by counties for 2008 and projected for 2035.

County	Population^*a*^ ≥ 45 years (%)^*b*^		CHD mortality (per 1,000)		CHD hospitalizations (per 1,000)	
	2008	2035	2008	2035	2008	2035
Los Angeles	3,321,703 (35.4)	5,189,815 (44.8)	3.7	5.0	9.1	10.7
Orange	1,085,184 (37.3)	1,501,496 (45.1)	2.6	4.4	6.8	9.7
Riverside	554,656 (33.0)	768,170 (40.6)	4.1	4.6	13.3	13.1
San Bernardino	466,992 (31.6)	672,435 (40.3)	2.2	5.4	8.1	13.8
Total	5,428,535 (35.1)	8,005,152 (43.3)	3.4	4.9	8.9	11.3
^***a***^Population is for the portion of the county that is within the South Coast Air Basin boundary, except for Orange County where the entire county is within the air basin. ^***b***^Percentage of the total (all ages) population.						

Annual average population-weighted traffic density was markedly skewed (see Supplemental Material, Figure S1a). The median 2008 traffic density was 14.4 (IQR = 3.9–30.1), after correcting for the fleet average PM_2.5_ emission reduction, and is projected to decrease to 11.6 (IQR = 4.1–22.3) in 2035 (from geometric mean of 10.8 in 2008 to 9.3 in 2035). In contrast, the proportion of the population living within 150 m from a freeway or 50 m from a major road is expected to increase from 8.3% to 10.9% from 2008 to 2035 (see Supplemental Material, Figure S1b). The mean (± SD) population-weighted EC level was 1.1 ± 0.4 μg/m^3^ in 2008 and is expected to decrease to 0.7 ± 0.3 μg/m^3^ in 2035 (see Supplemental Material, Figure S1c). The corresponding medians for the two periods were identical to the mean, 1.1 μg/m^3^ (IQR = 0.8–1.4) and 0.7 μg/m^3^ (IQR = 0.5–0.9), respectively. (The anticipated decrease is primarily due to the expected reduction of EC emissions from diesel-fueled vehicles.) The population mean PM_2.5_ exposure was 13.2 ± 4.2 μg/m^3^ in 2008, and is projected to decrease to 10.9 ± 3.7 μg/m^3^ in 2035 (see Supplemental Material, Figure S1d).

**Figure 2:**
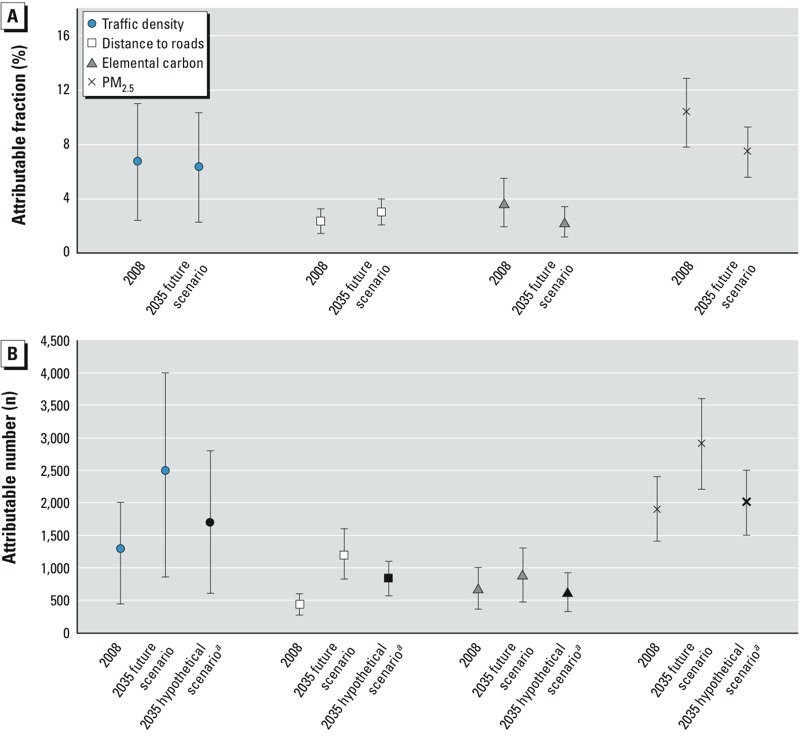
Population-attributable fractions (*A*) and population-attributable numbers (*B*) and 95% uncertainty intervals for coronary heart disease mortality in the South Coast Air Basin in 2008 and 2035,*^a^* attributed to traffic density within 300-m buffer from residence, residential distance to nearest freeway (≤ 150 m) or major road (≤ 50 m), elemental carbon, and regional PM_2.5_ above background levels of 1 for traffic density, 0% for proximity, 0.12 μg/m^3^ for EC, and 5.6 μg/m^3^ for PM_2.5_. Population-weighted mean exposures in 2008 and 2035 were 10.8 and 9.3 for traffic density, 1.1 and 0.7 μg/m^3^ for EC, and 13.2 and 10.9 μg/m^3^ for PM_2.5_, respectively. ***^a^***Population-attributable number that might be expected in 2035 if the age distribution of the 2035 population were the same as in 2008.

In 2008, an estimated 6.8% (95% UI: 2.4, 11.0) of the total CHD deaths among the population ≥ 45 years could be attributed to traffic density (Figure 2A). The PAF is expected to decrease to 6.4% (95% UI: 2.2, 10.3) in 2035, reflecting the expected decrease in population-weighted traffic density. The estimated 2008 PAF for residential distance of ≤ 150 m from freeways or ≤ 50 m from major roadways (2.4%; 95% UI: 1.4, 3.3) was smaller than the PAF for either traffic density or EC, but was projected to increase in 2035 to 3.1% (95% UI: 2.1, 4.0), reflecting the increase in proportion living close to major roadways. Based on estimated burden of EC exposure, 3.7% (95% UI: 1.9, 5.5) of the total CHD deaths in the ≥ 45 years age group in 2008 could have been prevented if the population-weighted mean EC exposure levels had been at the background level of 0.12 μg/m^3^ instead of 1.1 μg/m^3^. Decreasing population-weighted mean EC level is expected to result in decreased PAF to 2.3% in 2035 (95% UI: 1.2, 3.4). The estimated regional PM_2.5_ PAF was 10.4% (95% UI: 7.8, 12.9) in 2008 and is projected to fall to 7.5% (95% UI: 5.6, 9.3) in 2035.

Based on the NRAP PAFs for traffic density, an estimated 1,300 (95% UI: 440, 2,000) preventable deaths occurred in 2008, and 2,500 (95% UI: 860, 4,000) preventable deaths will occur in 2035 due to traffic density within 300 m of residences (Figure 2B). This large future increase is due to the projected increase in population, specifically to the disproportionate increase in the aging population at risk of CHD. This effect can be quantified using the hypothetical 2035 scenario in which the total population was increased as projected but was assigned the 2008 age distribution (essentially keeping the overall mortality rate unchanged). Under this hypothetical scenario, a much smaller number of deaths (1,700; 95% UI: 600, 2,800) would be attributable to traffic density. Based on the PAF for residential major road proximity (≤ 150 m from a freeway or ≤ 50 m from another major road), there were 430 preventable CHD deaths (95% UI: 270, 600) in 2008 and a projected 1,200 (95% UI: 820, 1,600) in 2035, compared with 830 (95% UI: 570, 1,100) that would be anticipated if the 2035 age distribution were the same as in 2008. For EC, 690 CHD deaths were attributable to exposure above background levels (95% UI: 360, 1,000) in 2008, about half of the estimated traffic density–attributable deaths but more than 1.5 times the major road proximity–attributable deaths. The EC-attributable deaths were also projected to increase less than that for traffic density, to 900 (95% UI: 470, 1,300) in 2035. Most of the estimated increase attributable to EC is due to the aging population structure rather than just the increase in population, which by itself would result in a small decrease in deaths to 630 (95% UI: 330, 920) because the population-weighted exposure is projected to decrease over time. About 1,900 deaths (95% UI: 1,400, 2,400) in 2008 were estimated to be attributable to regional PM_2.5_. A substantial increase to 2,900 (95% UI: 2,200, 3,600) is expected in 2035, despite a 25% decrease in PAF, due to the change in population and age distribution. In the hypothetical scenario in which only the population increases in 2035 without any change in age distribution, the PM_2.5_-attributable deaths would still increase to 2,000 (95% UI: 1,500, 2,500).

The overall pattern of changing exposure and NRAP-attributable CHD was generally similar across all SoCAB counties. Traffic density and EC levels were highest in Los Angeles County and lowest in Riverside County and are projected to decrease in all four counties from 2008 to 2035. (see Supplemental Material, Table S1). In contrast, the proportion living near a major road is projected to increase in all counties during the same period. Los Angeles County consistently had the highest estimated PAF and Riverside County the lowest based on each exposure in both 2008 and in 2035 (see Supplemental Material, Table S2). The estimated population-attributable number was consistently highest in Los Angeles (see Supplemental Material, Table S3), but traffic density–, EC-, and PM_2.5_-attributable numbers were each lowest in San Bernardino in 2008 and are expected to increase markedly by 2035, reflecting anticipated population increase under the compact urban development scenario.

The estimated PAF for CHD hospitalization attributable to EC exposure in the SoCAB was 1.9% (95% UI: 0.7, 3.1) in 2008, and is expected to decline to 1.2% (95% UI: 0.4, 1.9) in 2035 (see Supplemental Material, Table S4). The corresponding attributable number of hospitalizations was 920 (95% UI: 320, 1,500) for 2008 and is expected to increase slightly to 1,100 (95% UI: 380, 1,700) in 2035 after accounting for increases in population and hospitalization rate in an aging population. If the 2008 age distribution were applied to the 2035 population, the hypothetical number of hospitalizations might be expected to decrease to 840 (95% UI: 300, 1,400). The projected pattern of change over time in the county-specific estimates was generally similar to that for the entire SoCAB.

## Discussion

This study is one of the first risk assessments of CHD mortality and hospitalization attributable to NRAP markers and the first, to our knowledge, to project future estimates of the burden in a large metropolitan region. Estimates of the 2008 preventable CHD mortality due to NRAP among the ≥ 45 years population in the SoCAB varied from 2.4% (430 deaths), based on effects of residential proximity to a major road, to 6.8% (1,300 deaths), based on emissions-weighted traffic density. The traffic density–related burden in 2008 was about two-thirds the burden (10.4%, 1,900 deaths) attributable to regulated regional PM_2.5_. Thus, to the extent that NRAP and PM_2.5_ effects are independent, because regional PM_2.5_ does not characterize the sharp gradient in effects of the near-roadway pollutant mixture, a risk assessment based on PM_2.5_ alone is likely to be a substantial underestimate of the true pollution-attributable CHD mortality. The 2035 greenhouse gas reduction–planning scenario is projected to result in reduced population exposure and reduced PAF for PM_2.5_, traffic density, and EC (but not for residential proximity to major roadways). However, a surprising finding was that the attributable number of CHD deaths due both to PM_2.5_ and to each NRAP exposure, even under the optimistic planning scenario considered, is expected to increase substantially by 2035, largely due to vulnerability of an aging population. The proportion ≥ 65 years, at highest risk of CHD (Ford and Capewell 2007), is projected to double over the next two decades.

These results have important implications for health and urban planning policy. CHD accounts for most of the mortality attributable to PM_2.5_ levels in excess of the national standard (12 μg/m^3^) and therefore for the largest pollution-attributable annual economic costs, approximately $4.6 billion (adjusted to 2014 using the U.S. Bureau of Labor Statistics Consumer Price Index inflation calculator) (U.S. EPA 2013). Accounting for the effects of NRAP is likely to markedly increase estimates of economic cost of pollution. The increasing population-attributable number due to an aging population means that additional hospital beds and other health facilities will be needed for CHD treatment.

National air pollution regulations already adopted will have impacts over the next 20 years; examples include Tier-2 and Tier-3 vehicle standards (U.S. EPA 2014), and non-road diesel requirements (U.S. EPA 2004). These and the likely ongoing evolution of control technology requirements will contribute to reduced PM_2.5_ and EC emissions, and likely will reduce the impact of roadway proximity and traffic density (CRC 2013). We have not estimated the impact specific to greenhouse gas–reduction measures, independent of other pollution-reduction strategies. However, our results suggest that there are as yet unexploited opportunities for health benefits that would result from regulation of NRAP, and that additional health co-benefits could be obtained from the 2035 greenhouse gas reduction–planning process. The 2035 compact growth scenario used for this study will promote urban redevelopment with multi-family homes in corridors with good public transport to reduce reliance on private automobiles. The plan will promote investment in bicycling and walking infrastructure, and assumes that there will be increased vehicular fleet fuel efficiency and reduced emissions. However, if this planning scenario increases the population exposed to NRAP by placing people closer to busy roadways, they may be put at increased CHD risk, unless vehicle emissions were to decrease more substantially than currently anticipated. Variants on the planning scenario, such as policies to develop a zero- or close-to-zero-emission vehicle fleet, could optimize health co-benefits of greenhouse gas reduction. Another approach might be to encourage buffers between major traffic corridors and high-density development through zoning and other land use policies. Because markers for the NRAP mixture decrease sharply with distance to traffic, buffers of even a few 10s to 150 m are likely to decrease markedly the exposure and associated population burden of CHD morbidity and mortality, particularly for the elderly.

There are uncertainties in the estimates. The statistical uncertainty intervals are large. The estimated attributable burden also varied depending on the marker for NRAP. The 2008 traffic density–attributable CHD mortality was largest (6.8%) and the major roadway proximity-attributable mortality was smallest (2.4%). The traffic density burden was based on a CRF that used continuous exposure and accounted for volume of vehicles on all nearby roadways (Kan et al. 2008), and it was corrected for changing vehicles emissions over time. The smaller burden estimated from major roadway proximity might be expected because the CRF was based on a dichotomous classification that does not account for these factors (Gan et al. 2010), and therefore is the crudest surrogate for the NRAP mixture. Neither of these exposures accounts for meteorology and dispersion of a biologically relevant traffic pollutant such as EC, for which the number of attributable deaths in 2008 (*n* = 690) was between that for major roadway proximity exposure (*n* = 430) and traffic density (*n* = 1,300). EC had the smallest increase in 2035 NRAP-attributable mortality (which would be expected to decline if the population were not aging). The smaller EC-attributable burden in 2035 was due to an anticipated cleaner burning diesel vehicle fleet. EC- (and PM_2.5_-) attributable burden were also based on an assumption that no CHD effects would occur below background levels of 0.12 μg/m^3^ (EC) and 5.6 μg/m^3^ (PM_2.5_), which may have resulted in an underestimated burden.

EC is a toxicologically relevant component of particulate matter (Janssen et al. 2012) substantially influenced by pollution from heavy duty (diesel) vehicles in Southern California (Manchester-Neesvig et al. 2003). In this study, the estimated parcel level EC exposure used in calculating the burden accounted for the influence of meteorology on dispersion from local roadways, unlike the other two NRAP markers. However, the estimated EC exposure included both transported and local NRAP EC. Most (~ 90%) of the total EC exposure was regional and was common to all parcels in each 4 km × 4 km EC exposure grid. Thus, the estimated burden for EC reflected both regional and near-roadway effects, and EC effects may not be entirely independent of the burden assigned to the PM_2.5_ pollution, modeled solely on the regional scale. Therefore, the simple addition of the EC- and PM_2.5_-attributable events may overestimate the effect of these pollutants. It is difficult to assess the degree of such double counting, as there has been little study of the joint effects of exposure to EC and PM_2.5_ and the extent to which their effects are independent.

The uncertainty of the estimates based on future exposure scenario is likely to be greater than for the current estimates. For example, we corrected the traffic-density CRF based on an assumption that the effect of each vehicle exposure would decline in proportion to the decrease in fleet average PM_2.5_ vehicle emission rates per kilometer of travel since the original epidemiological study was conducted, equivalent to 15% from 2008 to 2035. The cruder traffic proximity exposure indicator was not adjusted for changes in vehicular emissions and therefore may overestimate the effect of this indicator. Alternatively, the proximity-attributable burden may reflect effects not scalable to changes in PM mass—for example, if the more toxic components of the mixture of fresh vehicular emissions changed to a different proportion than PM_2.5_ mass, or if components of resuspended road dust that might not change at all were the relevant hazard (Schwartz 1999). The uncorrected traffic density is actually projected to increase (by 6.5%) from 2008 to 2035, as is the population living near a major road [from 8.3% to 10.9% (see Supplemental Material, Figure S1b)]. Because the burden and costs of NRAP are large, additional research is warranted to reduce these sources of uncertainty.

Another important assumption is that the age-specific CHD rates will remain unchanged from 2008 to 2035. CHD mortality rates have fallen markedly over the last several decades in the United States (Ford et al. 2007) due to several factors. However, increased prevalence of obesity and its metabolic consequences are likely to slow this decline in CHD mortality rates and could potentially reverse them. Therefore, it is difficult to quantify the net impact of these trends on the estimates of NRAP-attributable burden of disease.

A limitation to the comparison of the NRAP- and PM_2.5_-attributable burden of CHD is that the original source CRFs were estimated for different age distributions. The PM_2.5_ CRF was developed for a population ≥ 30 years (Krewski et al. 2009), which we assigned to the population ≥ 45 years in order to be comparable to the population for the CRFs for all three indices of NRAP (Gan et al. 2010, 2011; Kan et al. 2008). PM_2.5_-attributable burden was considerably larger if applied to ≥ 30 years age group (3,100 fatal CHD events in 2008, e.g., compared with the 1,900 estimated based on the population ≥ 45 years). The larger estimate is generally consistent with other studies examining the burden of PM_2.5_-attributable CHD mortality statewide (CARB 2010). If the CRFs for NRAP were applied to the population ≥ 30 years, the estimated burden also increased markedly (data not shown). We have elected to use the common NRAP CRF age distribution for all estimates because NRAP is the exposure of primary interest. However, the estimated burden for both NRAP and PM_2.5_ restricted to the ≥ 45 year population is likely to be conservative.

Traffic-related noise has been associated with CHD, but whether it confounds, mediates, or interacts with near-roadway pollution is unclear (Fritschi et al. 2011). A recent review suggested that the two are likely independent risk factors of CHD (Davies and Kamp 2012), but this conclusion was based on only four studies. The CRFs we used were not adjusted for noise, so the near-roadway pollution-attributable burden could be independent or partially overlapping with the noise burden.

The health benefit from reduction in NRAP is unlikely to be limited to reductions in CHD mortality. We have not estimated burden of NRAP-attributable mortality associated with other outcomes, such as stroke and chronic obstructive pulmonary disease in the elderly, for which the causal relationships are less clear (HEI 2010). However, asthma and asthma exacerbation in children are likely caused by NRAP and have a large associated burden (Perez et al. 2012).

We calculated the PAF using the standard PAF formula (Equation 2). However, this estimate may be biased in the presence of confounding by characteristics in the study from which the CRF is derived if these covariates are not available for the target population (Darrow and Steenland 2011). There was little confounding of the CRFs for traffic density and EC by available covariates in the studies from which they were derived (Gan et al. 2011; Kan et al. 2008). However, the crude CRF associated with living near a major road was 1.69 and reduced to 1.29 after adjusting for confounders (age, sex, socioeconomic status, and co-morbidities) (Gan et al. 2010). These covariates are not available in our Southern California population data set. However, for a crude CRF/adjusted CRF of 1.69/1.29 (i.e., 1.3) and an exposure prevalence of 8.3% (the proportion of the 2008 SoCAB population living near a major road), our estimated traffic proximity PAF is likely to underestimate the true proximity PAF (Darrow and Steenland 2011).

Our results are likely to be relevant to other large North American cities with dispersed populations and high traffic volumes. We conclude that *a*) air pollution–attributable burden of CHD mortality may have been underestimated in most existing PM_2.5_-based risk assessments because they ignore NRAP effects, *b*) greenhouse gas–reduction planning offers additional opportunities for improving future cardiac health, if the NRAP risks are mitigated, and *c*) NRAP- (and PM_2.5_-) attributable CHD is likely to increase even if population exposure is reduced because of increased vulnerability of an aging population.

## Supplemental Material

(571 KB) PDF
