# Supplementary material for: Near-Roadway Air Pollution and Coronary Heart Disease: Burden of Disease and Potential Impact of a Greenhouse Gas Reduction Strategy in Southern California
Source: Environ Health Perspect. 2015 Jul 7;124(2):193–200. doi: 10.1289/ehp.1408865 (PMC4749075; doi:10.1289/ehp.1408865)
Supplement: (571 KB) PDF [file ehp.1408865.s001.acco.pdf]

*Environ Health Perspect*
DOI: 10.1289/ehp.1408865

**Note to Readers:** *EHP* strives to ensure that all journal content is accessible to all readers. However, some figures and Supplemental Material published in *EHP* articles may not conform to 508 standards due to the complexity of the information being presented. If you need assistance accessing journal content, please contact ehp508@niehs.nih.gov. Our staff will work with you to assess and meet your accessibility needs within 3 working days.

# Supplemental Material

# Near-Roadway Air Pollution and Coronary Heart Disease: Burden of Disease and Potential Impact of a Greenhouse Gas Reduction Strategy in Southern California

Rakesh Ghosh, Frederick Lurmann, Laura Perez, Bryan Penfold, Sylvia Brandt, John Wilson, Meredith Milet, Nino Künzli, and Rob McConnell

## Table of Contents

**Methods**

**Table S1.** Distribution of traffic density, elemental carbon and regional $PM_{2.5}$, and proportion living within ≤150m from a freeway or ≤50m from a major road among the population≥45 years of age in the South Coast Air Basin, by county, in 2008 and in 2035.

**Table S2.** Population attributable fraction (PAF) and 95% uncertainty interval (UI) for coronary heart disease mortality attributed to traffic density, residential proximity to roadways, elemental carbon and regional $PM_{2.5}$, for the South Coast Air Basin, by county, for 2008 and for 2035.

**Table S3.** Population attributable number and 95% uncertainty interval (UI) for coronary heart disease mortality for the South Coast Air Basin, by county, attributed to traffic density, residential proximity to roadways, elemental carbon and regional $PM_{2.5}$ for 2008 and for 2035.

**Table S4.** Population attributable fraction (PAF) and population attributable number with 95% uncertainty interval (UI) for coronary heart disease hospitalizations for SoCAB and for each county attributed to elemental carbon exposure.

**Figure S1.** Traffic density[a] within 300m buffer from residence (S2a), proportion living within ≤150m from a freeway or ≤50m from a major road (S2b), elemental carbon (2c) and regional $PM_{2.5}$[b] (2d) in the South Coast Air Basin in 2008 and in 2035. Boxes extend from the 25th to the 75th percentile, horizontal bar represent the median, whiskers extend 1.5 times the length of the interquartile range above and below the 75th and 25th percentiles, respectively, and outliers are represented as points. [a]Emission-weighted traffic density based on $PM_{2.5}$ reduction from 1990 to 2008 and 2035, which were -62.1% and -76.4%, respectively. [b]Double-headed arrow represents U.S. $PM_{2.5}$ National Ambient Air Quality Standard (NAAQS) of 12μg/m$^3$

**Supplemental References**

## Methods

The CRF for elemental carbon was obtained from a study in which black carbon absorption was measured (Gan et al. 2011). Elemental carbon data available for SoCAB was converted to black carbon absorption (Watson and Chow 2002) (for estimation of PAF and population attributable number) in units of $10^{-5}$/m, as follows:

$$Step\,1. \quad 10^{-5} \times Black\ Carbon_{absorption}(/m) = 0.8 \times Black\ Carbon_{mass}(\mu g / m^3)$$

$$Step\,2. \quad Black\ Carbon_{mass}\ (\mu g / m^3) = 0.79 \times Elemental\ Carbon_{mass}(\mu g / m^3)$$

$$Substituting\ Black\ Carbon_{mass}\ from\ step\ 2\ in\ step\ 1\ above$$

$$Step\,3. \quad 10^{-5} \times Black\ Carbon_{absorption}(/m) = 0.8 \times 0.79 \times Elemental\ Carbon_{mass}(\mu g / m^3)$$

**Table S1.** Distribution of traffic density, elemental carbon and regional $PM_{2.5}$, and proportion living within ≤150m from a freeway or ≤50m from a major road among the population≥45 years of age in the South Coast Air Basin, by county, in 2008 and in 2035.

| | 2008 | | 2035 | |
|---|---|---|---|---|
| **County** | **Mean ± SD or %** | **Median (Interquartile range)** | **Mean ± SD or %** | **Median (Interquartile range)** |
| Traffic density | | | | |
| Los Angeles | 35.0 ± 54.0 | 18.5 (6.9, 35.0) | 25.6 ± 36.6 | 13.8 (6.0, 25.1) |
| Orange | 28.9 ± 50.2 | 14.7 (3.8, 28.9) | 20.4 ± 32.8 | 10.9 (3.7, 20.0) |
| Riverside | 10.7 ± 24.7 | 3.0 (0.4, 10.4) | 11.6 ± 23.5 | 3.9 (0.2, 11.8) |
| San Bernardino | 16.9 ± 33.4 | 6.8 (1.3, 15.5) | 14.2 ± 25.0 | 6.3 (1.5, 13.3) |
| Residence within 150m from freeway or 50m from major road (%) | | | | |
| Los Angeles | 9.78 | | 12.79 | |
| Orange | 7.44 | | 8.99 | |
| Riverside | 2.75 | | 4.55 | |
| San Bernardino | 6.24 | | 7.69 | |
| Elemental Carbon (μg/m$^3$) | | | | |
| Los Angeles | 1.26 ± 0.39 | 1.32 (1.02, 1.51) | 0.81 (± 0.24) | 0.84 (0.68, 0.98) |
| Orange | 0.87 ± 0.28 | 0.91 (0.64, 1.08) | 0.57 (± 0.15) | 0.60 (0.45, 0.68) |
| Riverside | 0.59 ± 0.24 | 0.54 (0.41, 0.79) | 0.40 (± 0.15) | 0.37 (0.28, 0.51) |
| San Bernardino | 0.93 ± 0.32 | 0.93 (0.74, 1.09) | 0.62 (± 0.16) | 0.64 (0.51, 0.73) |
| Regional $PM_{2.5}$ (μg/m$^3$) | | | | |
| Los Angeles | 14.8 ± 4.1 | 15.9 (12.7, 17.4) | 12.4 ± 3.5 | 13.3 (10.6, 14.8) |
| Orange | 11.5 ± 2.7 | 12.2 (9.6, 13.6) | 9.1 ± 2.1 | 9.7 (7.5, 10.9) |
| Riverside | 8.5 ± 2.9 | 8.1 (6.3, 10.7) | 6.6 ± 2.2 | 6.1 (4.8, 8.3) |
| San Bernardino | 11.9 ± 3.0 | 12.4 (10.4, 13.8) | 9.3 ± 2.3 | 9.7 (7.8, 11.1) |

[a] Traffic density values were adjusted based on $PM_{2.5}$ reduction from 1990 to 2008 and to 2035, which were 62.1% and 76.4%, respectively.

**Table S2.** Population attributable fraction (PAF) and 95% uncertainty interval (UI) for coronary heart disease mortality attributed to traffic density, residential proximity to roadways, elemental carbon and regional $PM_{2.5}$, for the South Coast Air Basin, by county, for 2008 and for 2035.

| | 2008[a] | 2035[a] |
|---|---|---|
| **County** | **PAF (%)** | **PAF (%)** |
| Traffic density | | |
| Los Angeles | 7.56 (2.66, 12.21) | 6.98 (2.45, 11.30) |
| Orange | 6.74 (2.37, 10.92) | 6.12 (2.15, 9.94) |
| Riverside | 3.73 (1.30, 6.10) | 4.01 (1.40, 6.56) |
| San Bernardino | 5.03 (1.76, 8.20) | 4.84 (1.69, 7.89) |
| Residence within 150m from freeway or 50m from major road | | |
| Los Angeles | 2.76 (1.69, 3.81) | 3.58 (2.20, 4.94) |
| Orange | 2.11 (1.29, 2.93) | 2.54 (1.55, 3.52) |
| Riverside | 0.79 (0.48, 1.10) | 1.30 (0.79, 1.81) |
| San Bernardino | 1.78 (1.09, 2.47) | 2.18 (1.33, 3.02) |
| Elemental Carbon | | |
| Los Angeles | 4.39 (2.29, 6.45) | 2.69 (2.04, 3.34) |
| Orange | 2.90 (1.50, 4.28) | 2.19 (1.13, 3.24) |
| Riverside | 1.83 (0.94, 2.71) | 1.08 (0.56, 1.61) |
| San Bernardino | 3.12 (1.61, 4.61) | 1.92 (0.99, 2.84) |
| Regional $PM_{2.5}$ | | |
| Los Angeles | 12.42 (9.37, 15.37) | 9.34 (7.00, 11.62) |
| Orange | 8.11 (6.09, 10.09) | 4.88 (3.64, 6.10) |
| Riverside | 4.16 (3.10, 5.21) | 1.37 (1.02, 1.72) |
| San Bernardino | 8.68 (6.53, 10.78) | 5.21 (3.88, 6.52) |

[a] Estimates were for the difference between the 2008 or 2035 mean exposure (from Table S1) and background levels of 1 for traffic density, 0% for proximity, 0.12μg/$m^3$ for EC, and 5.6μg/$m^3$ for $PM_{2.5}$.

**Table S3.** Population attributable number and 95% uncertainty interval (UI) for coronary heart disease mortality for the South Coast Air Basin, by county, attributed to traffic density, residential proximity to roadways, elemental carbon and regional $PM_{2.5}$ for 2008 and for 2035.

| | **2008** [a] | **2035** [a] |
|---|---|---|
| **County** | **Attributable Number** | **Attributable Number** |
| Traffic density | | |
| Los Angeles | 930 (330, 1500) | 1800 (640, 2900) |
| Orange | 190 (70, 310) | 360 (130, 590) |
| Riverside | 90 (30, 140) | 140 (50, 230) |
| San Bernardino | 50 (20, 80) | 180 (60, 290) |
| Residence within 150m from freeway or 50m from major road | | |
| Los Angeles | 340 (210, 470) | 930 (570, 1300) |
| Orange | 60 (40, 80) | 170 (100, 230) |
| Riverside | 20 (10, 30) | 50 (30, 60) |
| San Bernardino | 20 (10, 30) | 80 (50, 110) |
| Elemental Carbon | | |
| Los Angeles | 540 (280, 790) | 700 (530, 870) |
| Orange | 80 (40, 120) | 150 (80, 210) |
| Riverside | 40 (20, 60) | 40 (20, 60) |
| San Bernardino | 30 (20, 50) | 70 (40, 100) |
| Regional $PM_{2.5}$ | | |
| Los Angeles | 1500 (1200, 1900) | 2400 (1800, 3000) |
| Orange | 230 (170, 290) | 320 (240, 400) |
| Riverside | 100 (70, 120) | 50 (40, 60) |
| San Bernardino | 90 (70, 110) | 190 (140, 240) |

[a] Estimates were for the difference between the 2008 or 2035 mean exposure (from Table S1) and background levels of 1 for traffic density, 0% for proximity, 0.12μg/$m^3$ for EC, and 5.6μg/$m^3$ for $PM_{2.5}$.

**Table S4.** Population attributable fraction (PAF) and population attributable number with 95% uncertainty interval (UI) for coronary heart disease hospitalizations for SoCAB and for each county attributed to elemental carbon exposure.

| | 2008 | | 2035 | |
|---|---|---|---|---|
| **County** | **PAF (%)** | **Attributable Number** | **PAF (%)** | **Attributable Number** |
| Los Angeles | 2.25 (0.78, 3.69) | 680 (240, 1100) | 1.37 (0.48, 2.25) | 760 (270, 130) |
| Orange | 1.48 (0.51, 2.44) | 110 (40, 180) | 1.12 (0.38, 1.85) | 160 (60, 270) |
| Riverside | 0.93 (0.33, 1.53) | 70 (20, 110) | 0.55 (0.20, 0.90) | 60 (20, 90) |
| San Bernardino | 1.59 (0.54, 2.63) | 60 (20, 100) | 0.98 (0.34, 1.62) | 90 (30, 150) |
| SoCAB | 1.90 (0.66, 3.12) | 920 (320, 1500) | 1.18 (0.42, 1.93) | 1100 (380, 1700) |

[a] Estimates were for the difference between the 2008 or 2035 mean exposure to EC (from Table S1) and background EC levels of 0.12μg/m$^3$. The SoCAB means for for 2008 and 2035 EC were 1.1 and 0.7μg/m$^3$, respectively.

## Figure Legend

**Figure S1.** Traffic density[a] within 300m buffer from residence (S2a), proportion living within ≤150m from a freeway or ≤50m from a major road (S2b), elemental carbon (2c) and regional $PM_{2.5}$[b] (2d) in the South Coast Air Basin in 2008 and in 2035. Boxes extend from the $25^{th}$ to the $75^{th}$ percentile, horizontal bar represent the median, whiskers extend 1.5 times the length of the interquartile range above and below the $75^{th}$ and $25^{th}$ percentiles, respectively, and outliers are represented as points. [a]Emission-weighted traffic density based on $PM_{2.5}$ reduction from 1990 to 2008 and 2035, which were -62.1% and -76.4%, respectively. [b]Double-headed arrow represents U.S. $PM_{2.5}$ National Ambient Air Quality Standard (NAAQS) of 12μg/$m^3$

Supplemental Material, Figure S1a.

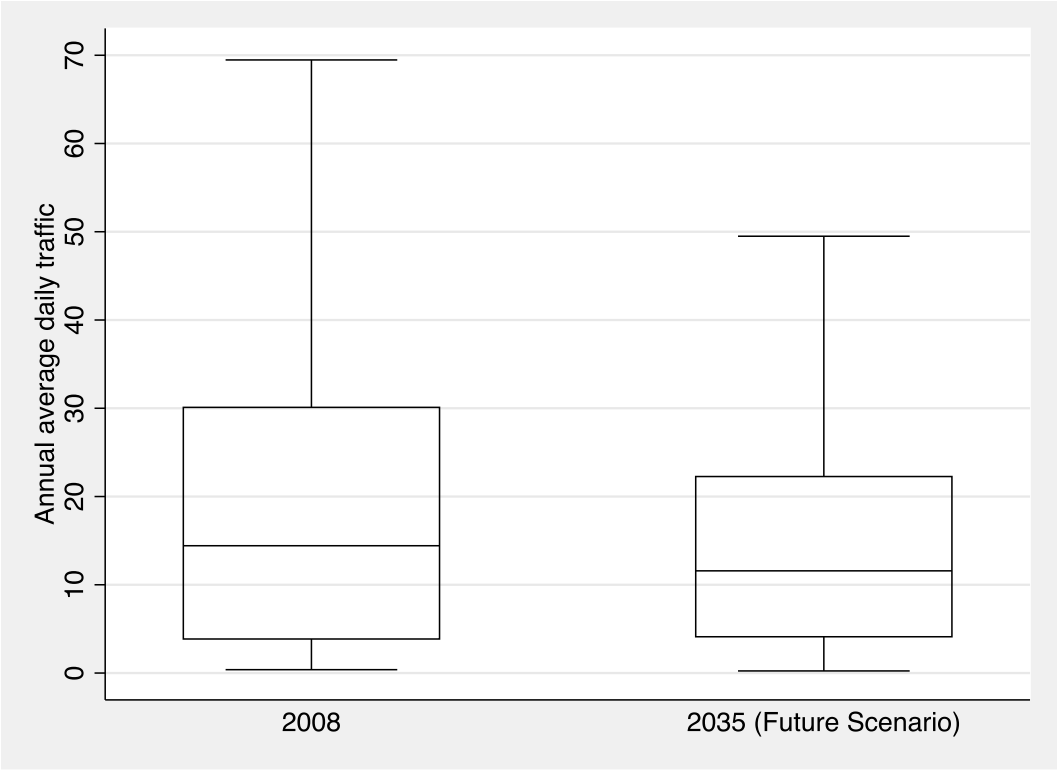


Figure S1b.

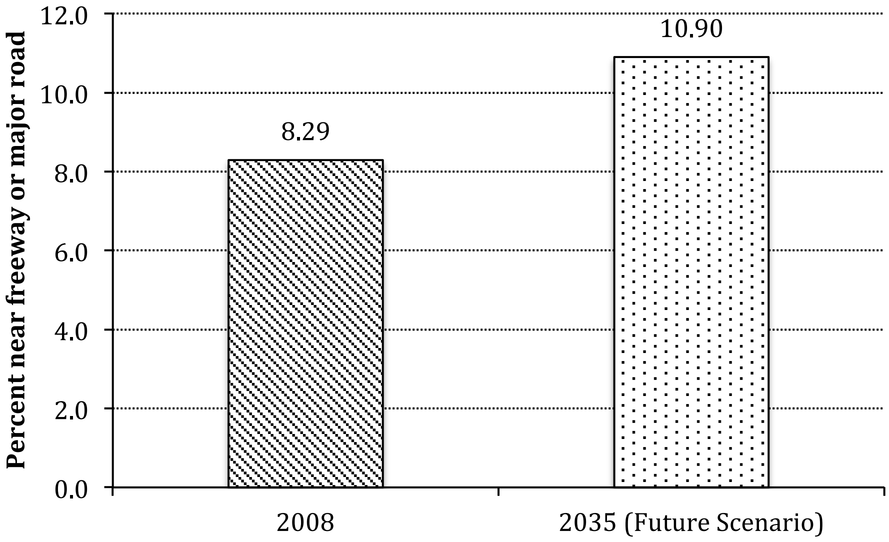

Figure S1c.

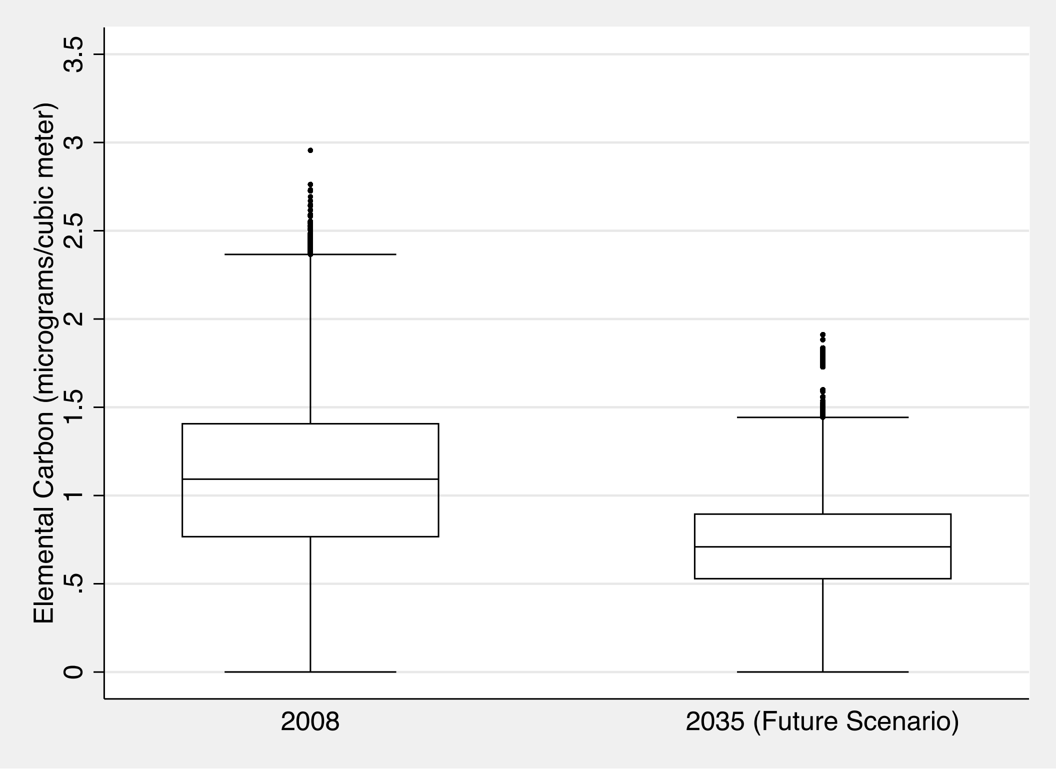

Elemental Carbon (micrograms/cubic meter)
0
.5
1
1.5
2
2.5
3
3.5
2008
2035 (Future Scenario)

Figure S1d.

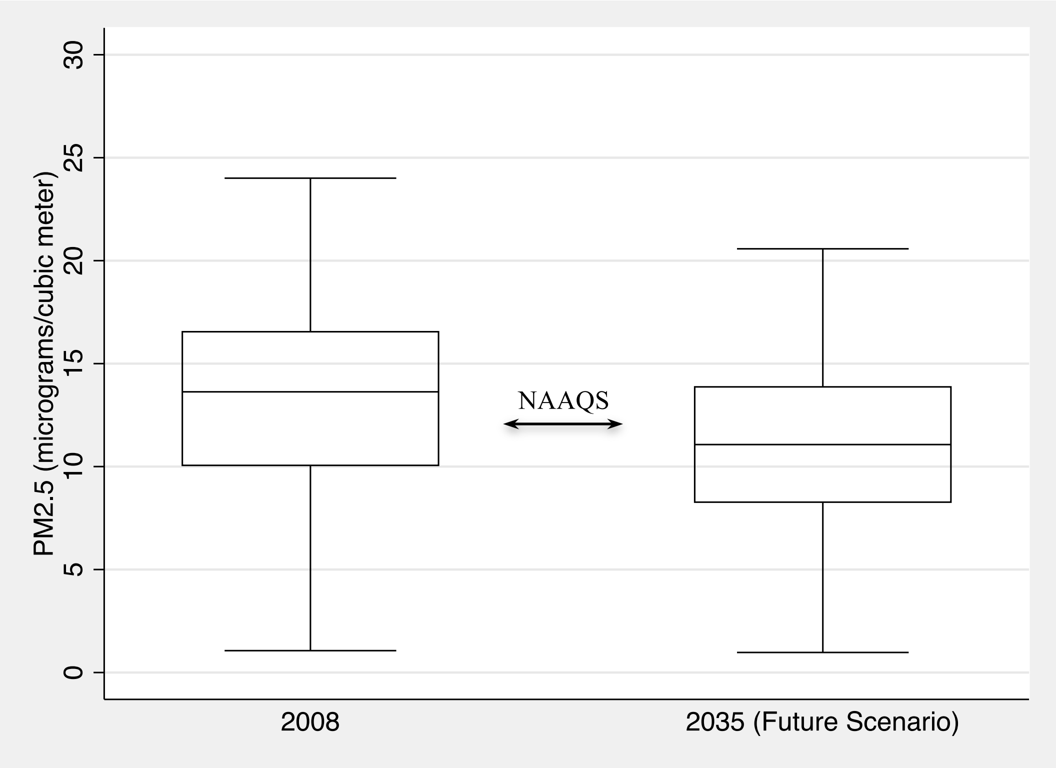
PM2.5 (micrograms/cubic meter)
0
5
10
15
20
25
30
NAAQS
2008
2035 (Future Scenario)

## Supplemental References

Gan WQ, Koehoorn M, Davies HW, Demers PA, Tamburic L, Brauer M. 2011. Long-term exposure to traffic-related air pollution and the risk of coronary heart disease hospitalization and mortality. Environ Health Perspect 119:501-507.

Watson JG, Chow JC. 2002. Comparison and evaluation of in situ and filter carbon measurements at the fresno supersite. Journal of Geophysical Reseasrch 107:8341.
